# Supplementary material for: Anomalous Angiogenesis in Retina
Source: Biomedicines. 2021 Feb 22;9(2):224. doi: 10.3390/biomedicines9020224 (PMC7927046; doi:10.3390/biomedicines9020224)
Supplement: Supplementary file 1 [file biomedicines-09-00224-s001.zip › supplementary.pdf]

SupplementaryMaterial for Anomalous Angiogenesis in Retina

Rocío Vega, Manuel Carretero, Luis L. Bonilla*
Gregorio Millán Institute, Fluid Dynamics, Nanoscience and Industrial Mathematics,
and Department of Mathematics, Universidad Carlos III de Madrid, 28911 Leganés, Spain

I. DETAILS OF THE COMPUTATIONAL MODEL

A. Cellular Potts model

We consider a square domain Ω of side L (in numerical simulations, $L = 230$ μm) having M nodes on each side. On Ω, there are $M \times M$ grid points and $(M-1)^2$ elementary squares (pixels) $\mathbf{x}$, each having an area $L^2/(M-1)^2$. Pixels $\mathbf{x}$ can belong to different cells or ECM, Σ_σ, and are labelled by their spin $\sigma(\mathbf{x})$ as follows:

$$\sigma(\mathbf{x}) = \begin{cases} 0, & \text{if } \mathbf{x} \in \Sigma_{\text{ECM}}; \\ 1, \dots,\ N_{\text{drusen}} + 1, & \text{if } \mathbf{x} \in \Sigma_{\text{BM}}; \\ N_{\text{drusen}} + 2 \dots,\ 2N_{\text{drusen}} + 1, & \text{if } \mathbf{x} \in \Sigma_{\text{drusen}}; \\ 2N_{\text{drusen}} + 2 \dots,\ 2N_{\text{drusen}} + 1 + N_{\text{RPE}}, & \text{if } \mathbf{x} \in \Sigma_{\text{RPE}}; \\ 2N_{\text{drusen}} + N_{\text{RPE}} + 2, \dots & \text{if } \mathbf{x} \in \Sigma_{\text{EC}}. \end{cases} \tag{1}$$

where N_{drusen} is the number of drusen and N_{RPE} is the number of RPE cells.

a. Energy functional. In the Hamiltonian of Eq. (1) in the main manuscript, we have

- The net variation of the durotaxis term H_{durot} is [1]

$$\Delta H_{\text{durot}} = -\rho_{\text{durot}}\, g(\mathbf{x}, \mathbf{x}')\, f(\mathbf{x}, \mathbf{x}'), \tag{2}$$

  where ρ_{durot} is a Potts parameter, $g(\mathbf{x}, \mathbf{x}') = 1$ for extensions and $g(\mathbf{x}, \mathbf{x}') = -1$ for retractions, and the function $f(\mathbf{x}, \mathbf{x}')$ depends on the solution of the elasticity equations as described in [3].

- The variation of the chemotaxis term H_{chem} is [2]

$$\Delta H_{\text{chem}} = -\rho_{\text{chem}}(\mathbf{x}, \mathbf{x}') \frac{C(\mathbf{x}') - C(\mathbf{x})}{1 + 0.3\, C(\mathbf{x})}, \tag{3}$$

  where C is the VEGF concentration in the corresponding pixel, given by Eqs. (2)-(5) of the main manuscript, and $\rho_{\text{chem}}(\mathbf{x}, \mathbf{x}') \geq 0$ is a Potts parameter that depends on the type of EC or ECM occupying pixels $\mathbf{x}$ and $\mathbf{x}'$. We have

$$\rho_{\text{chem}}(\mathbf{x}, \mathbf{x}') = \frac{\rho^0_{\text{chem}}}{\max_k D_k} \begin{cases} D_i, & \mathbf{x} \in \Sigma_i,\ \mathbf{x}' \in \Sigma_{\text{ECM}} \text{ or vice versa}, \\ \frac{D_i + D_j}{2}, & \mathbf{x} \in \Sigma_i,\ \mathbf{x}' \in \Sigma_j \text{ or vice versa}, \end{cases} \tag{4}$$

  where i and j are ECs. The positive constant ρ^0_{chem} measures the magnitude of chemotaxis. The level of Delta-4 determines the EC phenotype and, according to Eqs. (3) and (4), the strength of their chemotactic drive. Tip cells have a higher level of Delta-4 and, consequently by Eq. (4), they are more motile than stalk cells.

ρ_{area}(EC)	ρ_{area}(RPE)	ρ_{area}(DR)	ρ_{perim}(EC)	ρ_{perim}(RPE)	ρ_{perim}(DR)	ρ_{length}(EC)	ρ_{durot}	ρ^0_{chem}
25000	100000	750000	75	100	500	180	25	50000

TABLE I: Dimensionless Potts parameters corresponding to the area, perimeter, length, durotaxis and chemotaxis constraints (DR=drusen) [3].

The values of the Potts parameters are listed in Tables I and II from Ref. [3]. They are adjusted so that the terms in the net variation of the hamiltonian all have the same order. The perimeter contribution, absent in Refs. [1, 2], is small compared to the other terms in Eq. (1) of the main manuscript, so that it only affects the computations in extreme cases (e.g., extremely thin cells, thin cells that stick to the blood vessel). The sensitivity of this CPM to parameter values is discussed in Ref. [3].

---

* Corresponding author, email:bonilla@ing.uc3m.es

$\rho_{\text{adh}}^{\Sigma_\sigma,\Sigma_{\sigma'}}$	EC-EC	EC-ECM	EC-RPE	EC-DR	RPE-ECM	RPE-BM	RPE-DR	RPE-RPE	DR-ECM	DR-DR
Value	70-80	40	60	80	40	0-30	160	80-90	80	200

TABLE II: Dimensionless Potts parameters $\rho_{\text{adh}}^{\Sigma_\sigma,\Sigma_{\sigma'}}$ for adhesion (DR=drusen) [3].

B. Notch signaling pathway equations

The Notch signaling pathway is activated when the transmembrane receptor Notch belonging to a particular cell interacts with the transmembrane ligands Delta-4 or Jagged-1 belonging to its neighboring cell (trans-activation), thereby releasing the Notch intracellular domain (NICD). NICD then enters the nucleus and modulates the expression of many target genes of the Notch pathway, including both the ligands Delta and Jagged. However, when Notch of a cell interacts with Delta or Jagged belonging to the same cell, no NICD is produced; rather, both the receptor (Notch) and ligand (Delta or Jagged) are degraded (cis-inhibition) and therefore the signaling is not activated. These mechanisms are incorporated into model differential equations proposed in Ref. [4], and coupled to the CPM in Ref. [3]. Here, we describe these equations for a given cell i surrounded by other cells. At time t, let N_i, D_i, and J_i be the number of Notch, Delta-4, and Jagged-1 proteins in the ith cell, respectively. Similarly, let I_i, V_{Ri} and V_i be the number of NICD, VEGF receptor and VEGF molecules, respectively. These variables satisfy the following equations,

$$\frac{dN_i}{dt} = r_N H^S(I_i, \lambda_{I,N}) - \{[k_C D_i + k_T D_{\text{ext}}(i)]\, H^S(I_i, \lambda_{D,F}) + \gamma + [k_C J_i + k_T J_{\text{ext}}(i)]\, H^S(I_i, \lambda_{J,F})\}\, N_i, \tag{5a}$$

$$\frac{dD_i}{dt} = r_D H^S(I_i, \lambda_{I,D}) H^S(V_i, \lambda_{V,D}) - \left[k_C N_i H^S(I_i, \lambda_{D,F}) + k_T N_{\text{ext}}(i) + \gamma\right] D_i, \tag{5b}$$

$$\frac{dJ_i}{dt} = r_J H^S(I_i, \lambda_{I,J}) - \left[k_C N_i H^S(I_i, \lambda_{J,F}) + k_T N_{\text{ext}}(i) + \gamma\right] J_i, \tag{5c}$$

$$\frac{dI_i}{dt} = k_T N_i \left[H^S(I_i, \lambda_{D,F}) D_{\text{ext}}(i) + H^S(I_i, \lambda_{J,F}) J_{\text{ext}}(i)\right] - \gamma_S I_i, \tag{5d}$$

$$\frac{dV_{Ri}}{dt} = r_{VR} H^S(I_i, \lambda_{I,V_R}) - k_T V_{Ri} V_{\text{ext}}(i) - \gamma V_{Ri}, \tag{5e}$$

$$\frac{dV_i}{dt} = k_T V_{Ri} V_{\text{ext}}(i) - \gamma_S V_i. \tag{5f}$$

Here, r_N, r_D, r_J, and r_{VR}, are the production rates of N, D, J, and V_R, respectively. The cis-inhibition and trans-activation rates are k_C and k_T, respectively, whereas γ and γ_S are degradation rates for N, D, J, V_R and for I, V, respectively. These parameters, their representative values and units are listed in Table III. The shifted, excitatory and inhibitory Hill functions appearing in Eqs. (5) are:

$$H^S(\xi, \lambda_{\eta,\zeta}) = H^-(\xi) + \lambda_{\eta,\zeta} H^+(\xi), \tag{6a}$$

$$H^-(\xi) = \frac{1}{1 + \left(\frac{\xi}{\xi_0}\right)^{n_\zeta}}, \quad H^+(\xi) = 1 - H^-(\xi), \tag{6b}$$

where H^S is excitatory for $\lambda_{\eta,\zeta} > 1$ and inhibitory for $\lambda_{\eta,\zeta} \le 1$. In Eqs. (6), $\xi = V, I$, $\eta = I, V, D, J$, and $\zeta = N, D, J, V_R, F$ (the subscript F refers to Fringe, cf. [4]). The dimensionless parameters n_ζ and $\lambda_{\eta,\zeta}$ appearing in the Hill functions are listed in Table IV. We solved Eqs. (5) with zero initial conditions for all unknowns but the outcome of the simulations does not change if other initial conditions are used.

Parameter	r_N	r_D, r_J, r_{VR}	k_C	k_T	γ	γ_S
Value	1200	1000	5×10^{-4}	2.5×10^{-5}	0.1	0.5
Unit	molec/h	molec/h	(h molec)$^{-1}$	(h molec)$^{-1}$	h^{-1}	h^{-1}

TABLE III: Rates appearing in Eqs. (5).

If $j \in \langle i \rangle$ are the cells j sharing boundary of length $P_{i,j}$ with cell i, the number of $X = N, D, J$ molecules outside

Parameter	$\lambda_{I,N}$, $\lambda_{V,D}$, $\lambda_{I,J}$	$\lambda_{I,D}$, λ_{I,V_R}	$\lambda_{D,F}$	$\lambda_{J,F}$	n_N, n_D, n_V, n_{V_R}	n_J	n_F	I_0, V_0	χ_V
Value	2.0	0.0	3.0	0.3	2.0	5.0	1.0	200	1.0

TABLE IV: Dimensionless parameters appearing in the Hill functions. I_0 and V_0 are activation numbers of NICD and VEGF molecules, respectively, and χ_V is the conversion factor.

cell i is

$$X_{\text{ext}}(i) = \frac{1}{P_i} \sum_{j\in\langle i\rangle} P_{i,j} X_j. \tag{7}$$

The perimeter of cell i, P_i, minus $\sum_{j\in\langle i\rangle} P_{i,j}$ is the length of its boundary that is not shared with any other cell. Note that $X_{\text{ext}}(i)$ is simply the sum of all X_j if the lengths $P_{i,j}$ are all equal and $P_i = \sum_{j\in\langle i\rangle} P_{i,j}$ because the whole boundary of cell i is shared with other cells. As the cell moves and its boundaries fluctuate due to cellular Potts dynamics, the membrane protein levels of the neighboring cells interacting with the moving cell also vary. In this way, the production rates of the different proteins in a cell are directly influenced by the interactions with its neighborhood and, in particular, by the membrane fluctuations of the cell. $V_{\text{ext}}(i)$ is the number of VEGF molecules outside the ith cell that interact with VEGF receptor cells to produce VEGF molecules inside the ith cell. The external VEGF cells come from the continuum field $C(x, y, t)$, which diffuses from $x = L$. Let $\mathbf{x}_i$ be the pixel of the ith cell that is closer to the hypoxic region. The number of external VEGF molecules in that pixel is $C(\mathbf{x}_i, t)$ multiplied by the conversion factor $\chi_V = N_A L^2/[(M-1)^2 M_V]$, where M_V is the molecular weight of the VEGF molecules and N_A is the Avogadro number. We have used $\chi_V = 1$, which is representative of VEGF molecules with a large molecular weight. In the numerical simulation, C is known in the grid points and its value at a pixel should be the average value of the four grid points of the pixel. Since these values are quite similar, we adopt the value of C at the bottom left grid point of the pixel $\mathbf{x}_i$ as $C(\mathbf{x}_i, t)$.

Variable	N_i, D_i, J_i, N_{ext}, D_{ext}, J_{ext}	I_i	V_{Ri}	V_i	V_{ext}	t
Scale	$\sqrt{r_D/k_C}$	$(k_T r_D)/(k_C \gamma_S)$	r_{V_R}/γ	V_0	$6V_0$	$1/\sqrt{k_C r_D}$
Value	$\sqrt{2} \times 10^3$	10^2	10^4	2×10^2	12×10^2	$\sqrt{2}$
Unit	molec	molec	molec	molec	molec	h

TABLE V: Units for nondimensionalizing the Notch equations (5).

II. VIDEOS

Video 1: Left Column of Figure 7.
Video 2: Middle Column of Figure 7.

[1] Van Oers, R.F.M.; Rens, E.G.; La Valley, D.J.; Reinhart-King, C.A.; Merks, R.M.H. Mechanical cell-matrix feedback explains pairwise and collective endothelial cell behavior in vitro. *PLoS Comput. Biol.* **2014**, *10*(8), el003774.

[2] Bauer, A.L.; Jackson, T.L.; Jiang, Y. A Cell-Based Model Exhibiting Branching and Anastomosis during Tumor-Induced Angiogenesis. *Biophys. J.* **2007**, *92*, 3105–3121.

[3] Vega, R.; Carretero, M.; Travasso, R.D.M.; Bonilla, L.L. Notch signaling and taxis mechanims regulate early stage angiogenesis: A mathematical and computational model. *PLoS Comput. Biol.* **2020**, *16*(1), e1006919.

[4] Boareto, M.; Jolly, M.K. Ben-Jacob, E.; Onuchic, J.N. Jagged mediates differences in normal and tumor angiogenesis by affecting tip-stalk fate decision. *Proc. Natl. Acad. Sci. USA* **2015**, *112*, E3836–E3844.
